# Diallyl Sulfide Attenuation of Carcinogenesis in Mammary Epithelial Cells through the Inhibition of ROS Formation, and DNA Strand Breaks

**DOI:** 10.3390/biom11091313

**Published:** 2021-09-06

**Authors:** Selina F. Darling-Reed, Yasmeen Nkrumah-Elie, Dominique T. Ferguson, Hernan Flores-Rozas, Patricia Mendonca, Samia Messeha, Alicia Hudson, Ramesh B. Badisa, Syreeta L. Tilghman, Tracy Womble, Agnes Day, Marti Jett, Rasha Hammamieh, Karam F. A. Soliman

**Affiliations:** 1 Pharmaceutical Sciences Division, College of Pharmacy and Pharmaceutical Sciences, Florida A and M University, Tallahassee, FL 32307, USA; ynkrumahelie@gmail.com (Y.N.-E.); dominique3.ferguson@famu.edu (D.T.F.); hernan.floresrozas@famu.edu (H.F.-R.); patricia.mendonca@famu.edu (P.M.); samia.messeha@famu.edu (S.M.); alicia.hudson@famu.edu (A.H.); ramesh.badisa@famu.edu (R.B.B.); syreeta.tilghman@famu.edu (S.L.T.); tracy.womble@famu.edu (T.W.); karam.soliman@famu.edu (K.F.A.S.); 2 Department of Microbiology, College of Medicine, Howard University, Washington, DC 20059, USA; aday@howard.edu; 3 Medical Readiness Systems Biology, Walter Reed Army Institute of Research, Silver Spring, MD 20910, USA; marti.jett-tilton.civ@mail.mil (M.J.); rasha.hammamieh1.civ@mail.mil (R.H.)

**Keywords:** diallyl sulfide, benzo(a)pyrene, carcinogenesis, peroxide formation, DNA strand breaks, MCF-10A cells

## Abstract

Garlic has long been used medicinally for many diseases, including cancer. One of the active garlic components is diallyl sulfide (DAS), which prevents carcinogenesis and reduces the incidence rate of several cancers. In this study, non-cancerous MCF-10A cells were used as a model to investigate the effect of DAS on Benzo (a)pyrene (BaP)-induced cellular carcinogenesis. The cells were evaluated based on changes in proliferation, cell cycle arrest, the formation of peroxides, 8-hydroxy-2-deoxyguanosine (8-OHdG) levels, the generation of DNA strand breaks, and DNA Polymerase β (Pol β) expression. The results obtained indicate that when co-treated with BaP, DAS inhibited BaP-induced cell proliferation (*p* < 0.05) to levels similar to the negative control. BaP treatment results in a two-fold increase in the accumulation of cells in the G2/M-phase of the cell cycle, which is restored to baseline levels, similar to untreated cells and vehicle-treated cells, when pretreated with 6 μM and 60 μM DAS, respectively. Co-treatment with DAS (60 μM and 600 μM) inhibited BaP-induced reactive oxygen species (ROS) formation by 132% and 133%, respectively, as determined by the accumulation of H_2_O_2_ in the extracellular medium and an increase in 8-OHdG levels of treated cells. All DAS concentrations inhibited BaP-induced DNA strand breaks through co-treatment and pre-treatment methods at all time points evaluated. Co-Treatment with 60 μM DAS increased DNA Pol β expression in response to BaP-induced lipid peroxidation and oxidative DNA damage. These results indicate that DAS effectively inhibited BaP-induced cell proliferation, cell cycle transitions, ROS, and DNA damage in an MCF-10A cell line. These results provide more experimental evidence for garlic’s antitumor abilities and corroborate many epidemiological studies regarding the association between the increased intake of garlic and the reduced risk of several types of cancer.

## 1. Introduction

In the United States, in 2021, approximately 281,550 new diagnoses of breast cancer (BC) and 43,600 deaths are expected due to this disease [1]. Breast cancer is the foremost repeatedly diagnosed cancer in women, accounting for 30% of new female cancer cases [2]. Approximately 1 million breast cancer cases are diagnosed annually worldwide [3]. In breast cancer, many dysregulated signaling pathways are involved in the initiation and progression of the disease. Our recent results show that the pathway for the cytokine GRO-α/CXCL1 is dysregulated in triple-negative breast cancer (TNBC) [4]. Another related dysregulated pathway is the IƙBKE and MAPK1, indicating that GRO-α regulation is possibly through NFƙB and MAPK signaling pathways. Additionally, alterations found in the TNF superfamily receptor genes and the mRNA of receptors DR4 and DR5 expression binds to TNF-related apoptosis-induced ligand, a potent and specific stimulator of apoptosis in tumors [4]. Moreover, it is also known that cancer cells have increased reactive oxygen species (ROS) levels, leading to more carcinogenesis [4]. 

The cancer-preventive potential of natural products, especially phytochemicals, has become very appealing to study since epidemiological data indicated that consumption of plant-based foods is associated with a reduced risk of cancer [5]. Many phytochemicals contain various pharmacological properties, including anti-inflammatory, antioxidant, and anticancer properties. Several population studies show an association between the increased intake of garlic and reduced risk of certain cancers, including cancers of the stomach, colon, esophagus, pancreas, and breast [6]. Garlic with its organosulfur constituents has been studied extensively for its chemopreventive potential against cancer [6]. One of these compounds in garlic is diallyl sulfide (DAS). DAS is a garlic compound demonstrated to prevent carcinogenesis in vitro and reduce the incidence of stomach, colon, esophagus mammary, and lung cancers in vivo [7,8,9,10]. The concentration of DAS found in garlic ranges from 2 to 100 μg/g [7,11,12]. Shukla et al. [13] have shown that DAS reduced tumorigenesis and prolonged the life of Ehrlich ascites tumor-bearing mice. This effect is attributed to DAS-induced inhibition of cytochrome P450-2E1-mediated p-nitrophenol hydroxylation and N-demethylation of NDMA [14]. DAS inhibited aflatoxin-induced S9-dependent mutagenesis and the formation of its DNA-adducts [15], as well as the development of BaP-induced DNA adducts in human peripheral blood lymphocytes in vitro [16]. The chemopreventive potential of DAS in breast cancer was hypothesized due to its ability to attenuate the formation of diethylstilbestrol-induced mammary DNA adducts in female rats [17] and 2-amino-1-methyl-6-phenylimidazo[4,5-b] pyridine (PhIP) DNA strand breaks in breast epithelial cells [18,19]. 

We have previously reported [20,21] the effectiveness of two other garlic organosulfides, diallyl disulfide (DADS) and diallyl trisulfide (DATS), in the inhibition of BaP-induced breast cancer. The current study focuses on evaluating the effectiveness of DAS during the initiation phase, as indicated by biomarkers of early carcinogenic activity, including alterations to cell cycle, cell proliferation, and the formation of reactive oxygen species (ROS) and damaged DNA bases and DNA strand breaks. This study evaluates the first 24 h of BaP and DAS exposure of the normal breast epithelial cell line, MCF-10A, to further elucidate breast carcinogenesis initiation and determine its potential for inhibition.

## 2. Materials and Methods

### 2.1. Cell Line, Chemicals, and Reagents 

MCF-10A cells were purchased from American Type Culture Collection (ATCC, Manassas, VA, USA). Phenol red-free DMEM/F-12 media, horse serum, penicillin/streptomycin, antibiotic/antimycotic, human insulin (Novolin R), epidermal growth factor, trypsin-EDTA (10X), Hanks Balanced Salt Solution (HBSS), and Phosphate Buffered Saline (PBS) were purchased from Invitrogen (Carlsbad, CA, USA). Cholera toxin was purchased from Enzo Life Sciences (Plymouth Meeting, PA, USA). The CellTiter 96^®^ AQ_ueous_ One Solution Cell Proliferation Assay was purchased from Promega (Madison, WI, USA). The Bromodeoxyuridine Cell Proliferation (Chemiluminescent) Assay kit was purchased from Cell Signaling Technology (Danvers, MA, USA). The EpiQuik 8-OHdG DNA Damage Quantification Direct Kit (Colorimetric) was purchased from EpiGentek (Farmingdale, NY, USA). The Qiagen Genomic-tip 20/G, Genomic DNA buffer set, and proteinase k were purchased from Qiagen (Germantown, MD, USA). benzo(a)pyrene (BaP), diallyl sulfide (DAS), PeroxiDetect^TM^ Kit, and all other chemicals were purchased from Sigma-Aldrich (St. Louis, MO, USA). Antibodies were purchased from Cell Signaling (GAPDH (D16H11) Rabbit mAb; #5174S) and Abcam (DNA Polymerase β; ab26343).

### 2.2. Cell Culture

MCF-10A cells were cultured in DMEM/F12 media supplemented with horse serum (5%), human insulin (10 μg/mL), cholera toxin (100 ng/mL), epidermal growth factor (20 ng/mL), hydrocortisone (0.5 μg/mL), and penicillin-streptomycin. Cells were grown to 90%–100% confluence in an incubator at 37 °C under a humidified atmosphere of 5% CO_2_. The supplemented media was changed every 2–3 days, and the cells were sub-cultured every 5–7 days.

### 2.3. Cell Treatments 

This study was conducted using the established epithelial cell model, MCF-10A, a non-tumorigenic cell line. The cells were plated and treated according to two treatment groups: (1) DAS pre-treatment (PreTx), in which cells were treated with 6, 60, or 600 μM of DAS for 4 h prior to the addition of 1 μM BaP, or (2) BaP and DAS co-treatments (CoTx), in which the cells were treated concurrently with both 1.0 μM BaP and 6, 60, or 600 μM of DAS. All treatments were prepared in 0.1% DMSO under low light conditions. Treatments were added to cells in serum-free media. Following treatment, the cells were incubated for 3, 6, 12, or 24 h in a 37 °C, 5% CO_2_ humidified incubator. Untreated cells and 0.1% DMSO vehicle controls were prepared for all of the experiments.

### 2.4. Cell Harvesting

After treatment, cells were washed twice with HBSS for 2 min at room temperature to remove the serum. The adherent cells were trypsinized for detachment using trypsin-EDTA in HBSS for 10–15 min in a 37 °C, 5% CO_2_ humidified incubator. Following neutralization of trypsin with an equivalent amount of supplemented media, the cells were pipetted into 15 mL centrifuge tubes and centrifuged at 2500 rpm for 2 min. The media was removed, and the cells were resuspended in Mg^2+^ or Ca^2+^ free PBS.

### 2.5. MTS Assay for Determination of Cell Viability 

Untreated MCF-10A cells (2 × 10^4^/well) were plated into 96 well flat bottom plate (100 μL/well) in serum-free media and allowed to adhere overnight. The media was aspirated from each well, and 100 μL of each treatment, described above, prepared in supplemented serum-free media, was applied to the cells in replicates of n = 8. Following incubation for 3, 6, 12, or 24 h, cell viability was carried out and assessed according to the manufacturer’s protocol for the CellTiter 96^®^ AQ_ueous_ One Solution MTS Assay and previous studies [20,21]. Absorbance was measured using a PowerWave X-340 96-well Plate Reader (Bio-Tek Instruments, Inc., Winooski, VT, USA). The results were normalized to the control (cells only), represented as 100% cell viability.

### 2.6. Bromodeoxyuridine (BrdU) Cell Proliferation (Chemiluminescent) Assay

To measure cell proliferation in MCF-10A cells, the BrdU cell proliferation assay was conducted according to the bromodeoxyuridine cell proliferation kit instructions. Briefly, after the assay kit reagents were prepared for BrdU incorporation, cells were plated in a 96-well plate at a 50,000 cell/well density in replicates of N = 8 and incubated with cell treatments described above for 12 and 24 h. All cell treatments will be normalized to the control (0.1% DMSO). Cells were incubated for 24 h with BrdU followed by fixation, secondary antibody labeling, and luminol enhancer solution. Luminescence was measured at 450 nm using a Biotek Synergy H1 microplate reader. 

### 2.7. Flow Cytometry for Analysis of Cell Cycle 

MCF-10A cells were treated as described above. The cells were cultured in a 75 cm^2^ culture flask until they reached 40–50% confluency and were fixed. Staining and flow cytometry analysis was conducted as previously described [20,21] using the Becton-Dickinson FACSCalibur Flow Cytometer (Becton Dickinson Biosciences, Franklin Lakes, NJ, USA). Phase distributions were determined by ModFit software (Verity Software House, Topsham, ME, USA).

### 2.8. Aqueous Peroxide Detection 

Protocol for aqueous peroxide determination is according to the method described in prior studies [20] and the manufacturer instructions for the PeroxiDetect Kit to determine Aqueous Hydroperoxides. As a positive control, 0.1% H_2_O_2_ was used.

### 2.9. Single Cell Gel Electrophoresis (Comet Assay) 

MCF-10A cells were treated at 100% confluence as described above in 75 cm^2^ culture plates. The Comet Assay was performed according to methods described in prior studies [19,20,21] (each sample in triplicate). Cells were analyzed by Kinetic Imaging (Kinetic Imaging Ltd., Merseyside, UK) and Komet 5.5 software (And/or Technology, Belfast, Northern Ireland) to determine the mean olive tail moment (MOTM) for each cell, as a measure of DNA damage.

#### 2.10. 8-Hydroxy-2-deoxyguanosine (8-OHdG) Detection

8-OHdG, a well-known marker of oxidative stress, was measured in treated MCF-10A cells using the EpiQuik 8-OHdG DNA Damage Quantification Direct Kit (Colorimetric). Briefly, genomic DNA was extracted (using Qiagen Genomic-tips 20/G, Genomic DNA buffer set, and proteinase k) from previously treated cells and stored at −20 °C for later use in measuring 8-OHdG expression. To measure 8-OHdG, DNA was bound to a 96-well flat-bottom plate followed by a wash and the addition of the capture antibody. After the second wash, the detection antibody and enhancer solution were added. The color-developing solution was added to measure at an absorbance of 450 nm using the Biotek Synergy HTX Multi-mode microplate reader. 

### 2.11. Capillary Electrophoresis Western Analysis

Cell pellets corresponded to control (cells + DMSO), BaP-treated (1 µM), and co-treated (DAS 60 µM and 600 µM combined with BaP 1 µM) cells after 24-h treatment. A protease inhibitor cocktail was mixed with the lysis buffer, and the combination was added to each pellet. The total protein expression was determined using Western analysis. Samples containing 0.6 mg/mL of protein and 1:125 dilution of primary antibody were used. The microplate was loaded according to ProteinSimple’s protocol and placed in the instrument, along with the capillary responsible for the reaction. DNA Polymerase β (Abcam #ab26343) and loading control GAPDH (D16H11; Cell Signaling #5174S) were the specific primary antibodies tested. The protein was identified, the chemiluminescence reaction was determined, and the digital blot image was taken. 

### 2.12. Statistical Analysis 

GraphPad Prism 9.0 software (San Diego, CA, USA) was used to analyze all of the data using one-way analysis of variance (ANOVA) with Bonferroni’s Multiple Comparison Test. The results are displayed as the average values ± SEM to determine significant differences (*p* < 0.05) between the treatment groups and the vehicle (DMSO) and BaP only controls. 

## 3. Results

### 3.1. DAS Inhibits BaP-Induced Cell Proliferation Using MTS and BrdU Proliferation Assays

**Figure 1:**
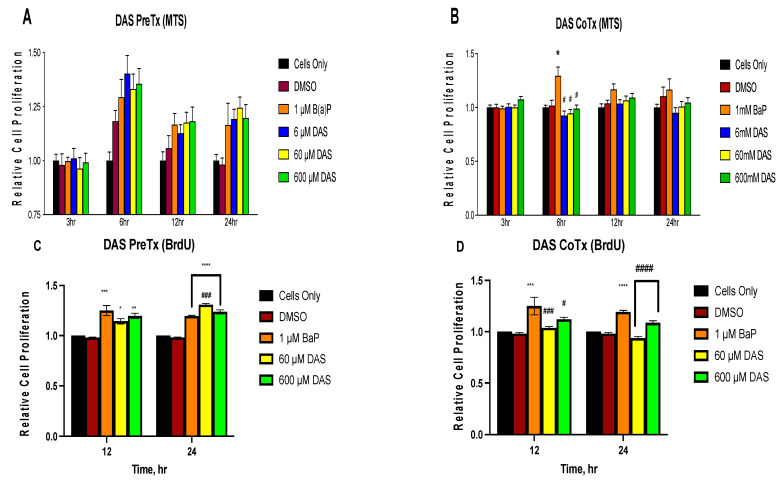
Relative cell proliferation (fold changes) of cells treated with DAS and BaP. MCF-10A cells were treated with 1 μM BaP only, PreTx (**A**,**C**) or CoTx (**B**,**D**) with DAS (6, 60, and 600 μM) during BaP (1 μM) exposure for 3, 6, 12, and 24 h. Cell proliferation was assessed using MTS (**A**,**B**) and BrdU incorporation assay (**C**,**D**). The absorbance of each treatment group was normalized to the cells only control. The control is expressed as one-fold growth for each time point. The graph represents the average relative cell proliferation of triplicate values ± SEM. (* indicates *p* < 0.05, ** *p* < 0.01, *** *p* < 0.001, **** *p* < 0.0001 significant difference compared to DMSO control, and # *p* < 0.05, ### indicates *p* < 0.001, #### indicates *p* < 0.0001 significant difference when compared to 1 μM BaP treatment using Bonferroni’s multiple comparison correction Test).

**Figure 2:**
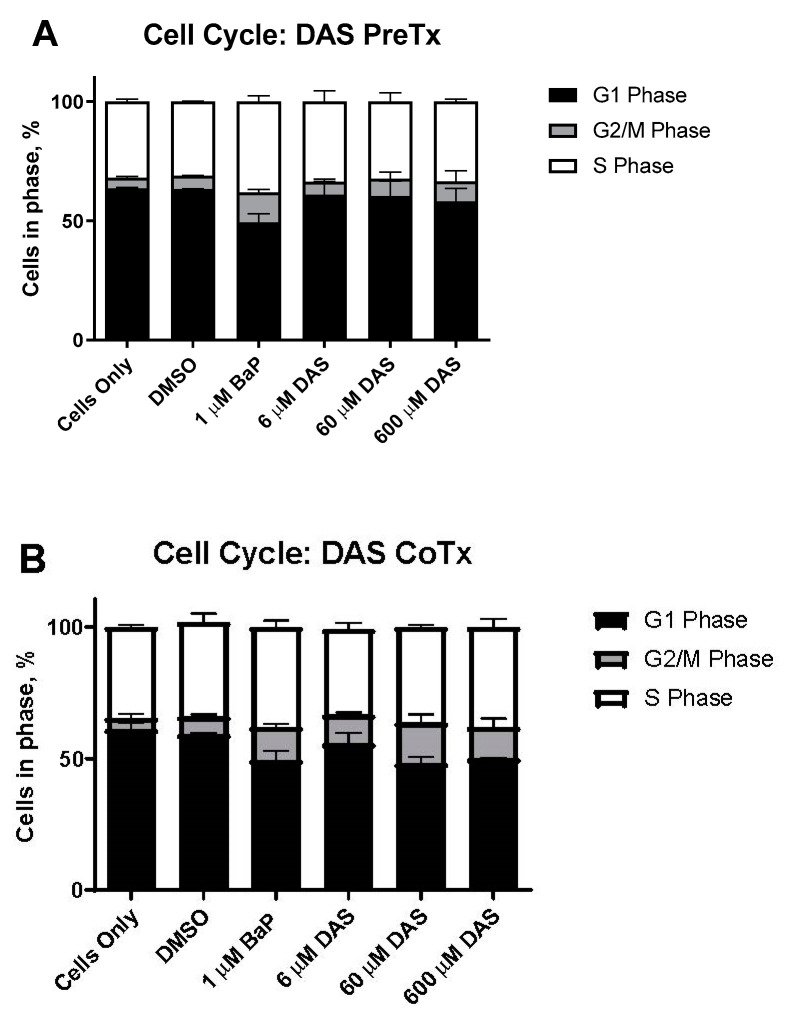
DAS inhibits BaP-induced G2/M cell cycle transition. The results of cell cycle analysis of MCF-10A were either pretreated with DAS (6, 60, and 600 μM) followed by treatment with 1 μM BaP for 24 h (**A**) or co-treated with DAS and BaP for 24 h (**B**). The cells were fixed in ethanol and analyzed by propidium iodide staining for flow cytometry. The values represent the average percent of cells in each phase, G1, G2/M, and S, for N = 3, ±SEM.

Changes in cell proliferation of MCF-10A cells treated with DAS and BaP were evaluated using the MTS assay. BaP caused a significant (*p* < 0.05) increase in cell proliferation after 6 h of exposure, followed by a slight decrease in cell proliferation at 12 and 24 h of BaP exposure relative to the vehicle control (Figure 1A,B). DAS CoTx reduced cell proliferation by 18.6%, 12.7%, and 14.4%, (for 6, 60, and 600 μM, respectively at 6 h) respectively, relative to the vehicle control (Figure 1B), while the DAS PreTx caused a slight increase (not significant) in cell proliferation relative BaP alone or vehicle control (Figure 1A). After 12 and 24 h of exposure, DAS CoTx also inhibited the nominal increase in BaP-induced cell proliferation. The most effective inhibition of proliferation, relative to the vehicle control, was achieved with 6 μM of DAS (Figure 1B). In the DAS PreTx group, however, DAS was not effective. Changes in cell proliferation of MCF-10A cells treated with BaP and DAS for 12 and 24 h observed in the MTS assay were validated using the BrdU proliferation assay. This assay was chosen since it targets DNA through BrdU measurement, which incorporates into proliferating DNA rather than the assessment of mitochondrial function (through the formation of formazan crystals) in the MTS assay. Exposure to 1 μM BaP caused a significant increase in cell proliferation at 12 (*p* < 0.001) and 24 (*p* < 0.0001) hours when compared to the DMSO control (Figure 1C,D). Similarly, there was a significant increase in cell proliferation following DAS PreTx (60 (*p* < 0.05) and 600 (*p* < 0.01) μM) when compared to control (Figure 1C). While 60 and 600 μM DAS PreTx caused no changes at 12 h, 60 μM DAS caused a significant increase in cell proliferation at 24 h compared to 1 μM BaP (Figure 1D). At 12 h of exposure, 60 (*p* < 0.001) and 600 (*p* < 0.05) μM CoTx caused a significant decrease in cell proliferation with a more significant decrease of 17.3% and 8.6% in 60 and 600 μM DAS CoTx (*p* < 0.0001), respectively, following 24 h of exposure when compared to 1 μM BaP (Figure 2B).

### 3.2. DAS Prevents BaP-Exposed MCF-10A Cells from Arresting at S and G2/M

**Figure 3:**
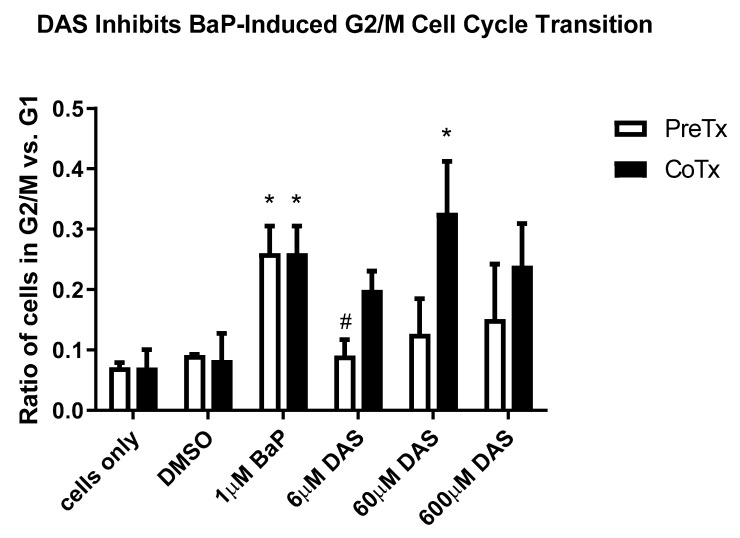
The ratio of cells in G2/M and G1 phases resulting from DAS Pre- and CoTx. The average values for the N = 3 treatments for the cell cycle were compared for the PreTx and CoTx. The values represent the G2/M phase divided by the corresponding G1 phase for N = 3, ±SEM (* indicates a *p* < 0.05 significant difference from DMSO control, and # indicates a *p* < 0.05 significant difference from the 1 μM BaP treatment) at inhibiting the modest BaP-induced increases in cell proliferation after 12 and 24 h of exposure (Figure 1A).

Cell cycle analysis was performed on MCF-10A cells to evaluate them for S and G2/M arrest following BaP and DAS treatment. MCF-10A cells accumulated in the S and G2/M phases after 24 h of exposure to increasing concentrations (0–2 μM) of BaP. BaP (1 μM) induced a 119% increase in cells in the G2/M phase compared to the vehicle control (Figure 2A,B). BaP-induced alterations in the cell cycle occurred in these cells following 24-h DAS PreTx and CoTx exposure. At the lowest concentration DAS (6 μM) inhibited the BaP-induced increase in MCF-10A cells accumulation in G2/M-phase by 128%, and the 60 and 600 μM concentrations inhibited the cell cycle transition by 94% and 83%, respectively. Comparing the ratio of cells in G2/M versus the G1 phase (Figure 3) indicated an apparent increase in cells treated with BaP compared to the control. Simultaneously, the DAS PreTx was more effective than DAS CoTx at reducing the ratio of cells in G2/M versus the G1 phase compared to the BaP treatment.

### 3.3. Reduction in the Accumulation of ROS in BaP-Treated Cells by DAS

**Figure 4:**
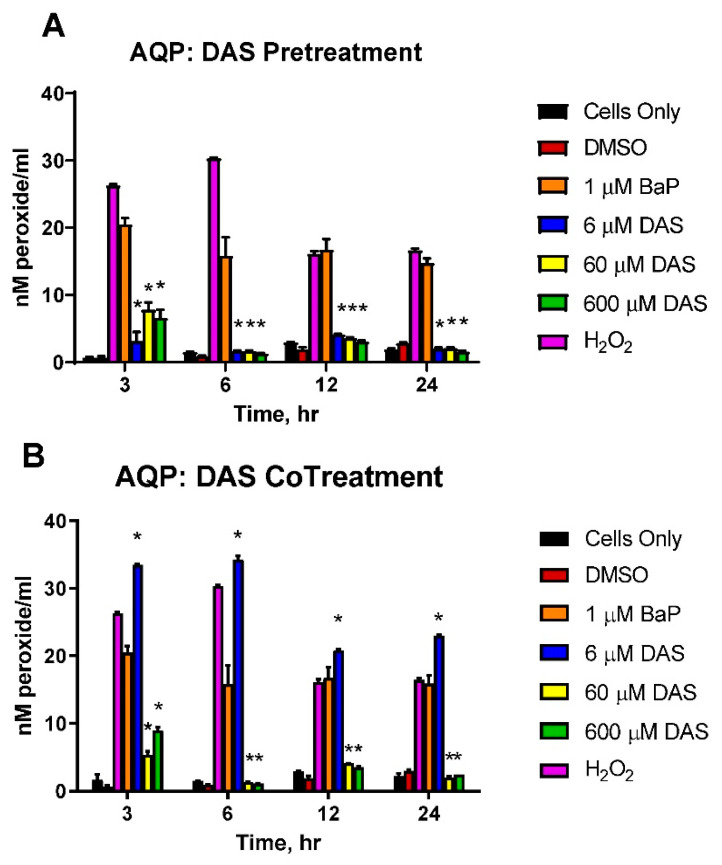
DAS inhibition of BaP-induced Aqueous Peroxides. The cells analyzed for Aqueous Peroxides were either pre-treated (**A**) with DAS followed by BaP or co-treated (**B**) with DAS and BaP for 3, 6, 12, and 24 h. The average values for replicates of N = 3 were compared to the cells pretreated (**A**) or co-treated (**B**) with DAS (6, 60, and 600 μM) and BaP (1 μM) (* indicates a *p* < 0.05 significant difference from DMSO control) in triplicate, ±SEM. As a positive control, 0.1% Hydrogen peroxide was used.

The levels of aqueous peroxides (AQP) resulting from PreTx and CoTx with DAS and BaP are compared in Figure 3. BaP induced a significant increase in AQP production, which peaked at 6 h; however, DAS PreTx inhibited such production at all points. For 3, 6, 12, and 24 h, the 6 μM DAS PreTx suppressed BaP-induced AQP by 87.5%, 95.0%, 84.9%, and 107.5%, respectively; the 60 μM DAS PreTx resulted in 64.0%, 95.1%, 88.7%, and 106.6% reduction, respectively (Figure 4A). The 600 μM DAS PreTx caused a 70.1%, 97.6%, 92.2%, and 110.8% respective suppression. Using the above listed time points, 6 μM DAS CoTx indicated an additive effect, increasing AQP by 65.5%, 123.9%, 27.3%, and 69.2% compared to control, respectively. Whereas inhibition of AQP occurred by 76.3%, 97.0%, 85.1%, and 107.2% following 60 μM DAS CoTx and by 57.8%, 98.6%, 89.0% and 103.3% following 600 μM DAS CoTx, respectively (Figure 4B). These results indicate PreTx was more effective in inhibiting BaP-induced ROS formation as detected by aqueous peroxides production. 

### 3.4. Inhibition of BaP-Induced DNA Damage by DAS in MCF-10A Cells

**Figure 5:**
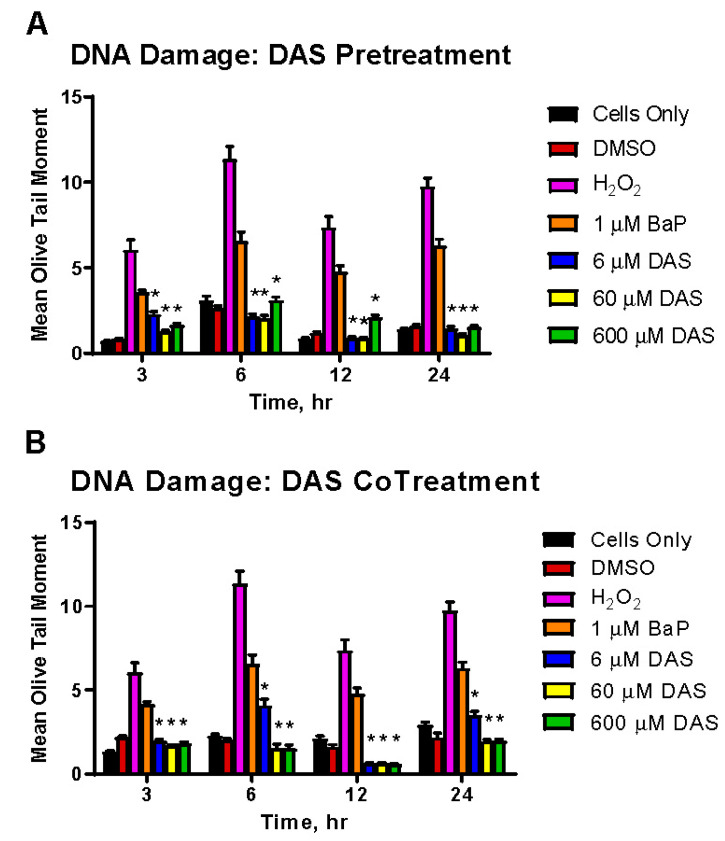
DAS inhibition of BaP-induced DNA strand breaks. The Comet assay demonstrates DAS attenuation of BaP-induced DNA strand breaks through PreTx(**A**) and CoTx(**B**). BaP (1 μM) treated cells were either pretreated or co-treated with DAS (6, 60, and 600 μM) for 3, 6, 12, and 24 h. The results represent the mean Olive tail moment as an indicator and quantifier of DNA damage, ±SEM for 150 cells for N = 3 (* indicates a *p* < 0.05 significant difference from 1 μM BaP only).

BaP induces significant DNA strand breaks, as demonstrated by the mean olive tail moment (MOTM; Figure 5). To test whether DAS could attenuate BaP-induced DNA strand breaks, we performed COMET assays on MCF-10A cells using either the PreTx or CoTx procedures with 6, 60, or 600 μM of DAS. DAS PreTx significantly attenuated BaP-induced DNA damage at 3, 6, 12, and 24 h (range of 47.2–114.5%) with the most profound effect observed at 6 h (112.4%, 114.5%, and 87.8%, for 6, 60, or 600 μM of DAS, respectively) (Figure 5A). While a significant attenuation of BaP-induced DNA damage was observed in the DAS CoTx group at 3, 6, 12, and 24 h (range of 54.6–133.0%), this attenuation was most prominent at 12 h (130.2%, 131.5%, and 133.0%, for 6, 60, or 600 μM of DAS, respectively) (Figure 5B). Damaged DNA decreases in all treatment groups by 24 h, including untreated control, possibly indicating a reduction in damage or both an increase in DNA repair and reduction of damage. 

### 3.5. Inhibition of BaP-Induced Oxidative (8-OHdG) DNA Damage by DAS in MCF-10A Cells

**Figure 6:**
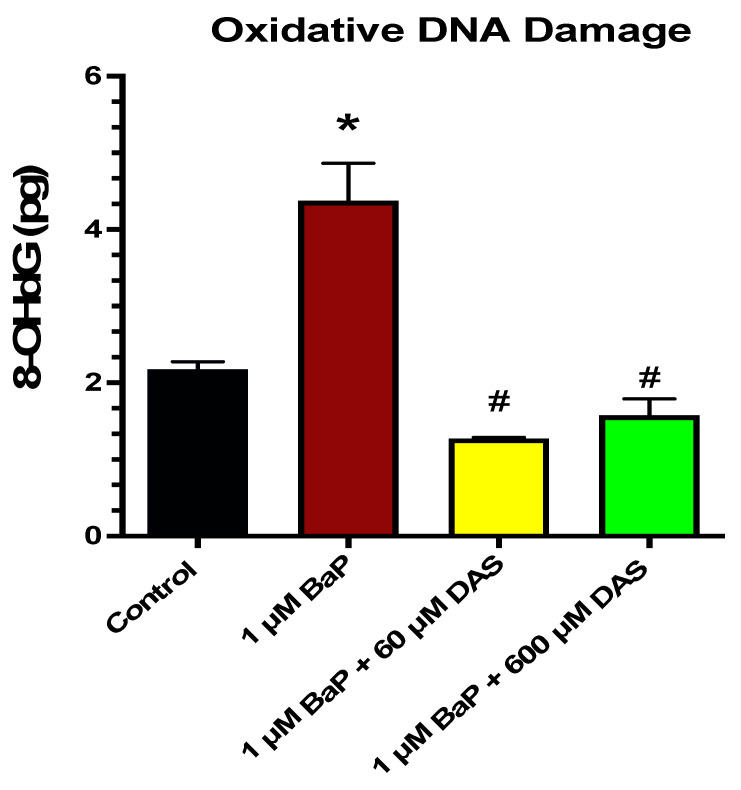
DAS and/or BaP effect on oxidative DNA damage in Normal Breast Epithelial Cells (MCF-10A) determined by 8-OHdG DNA damage quantification. 8-OHdG is an indicator of oxidative DNA damage. MCF-10A cells were exposed to 1 μM BaP and/or (60 and 600 μM) DAS CoTx for 24 h. The results represent pg of 8-OHdG as an indicator of oxidative DNA damage. The data points are presented as the mean ±SEM of three replicates. The significance of the difference was analyzed using an unpaired *t*-test to compare 8-OHdG expression vs. control (* *p* < 0.05) or vs. treated cells (^#^ *p* < 0.05).

Oxidative DNA damage was measure using the Epiquik 8-OHdG DNA Damage Quantification Direct Kit. This assay employs the modified nucleoside base, 8-hydroxy-2-deoxyguanosine (8-OHdG), an oxidized derivative of deoxyguanosine generated by singlet oxygen, hydroxyl radicals, and one-electron oxidants in cellular DNA, as a sensitive oxidative DNA damage and oxidative stress marker. BaP caused a significant increase in 8-OHdG when compared by control, while both 60 and 600 μM DAS CoTx caused a significant decrease in 8-OHdG when compared to 1 μM BaP (Figure 6). 

### 3.6. Increase in DNA Polymerase β (POLβ) Protein Expression by DAS in BaP Treated MCF-10A Cells

**Figure 7:**
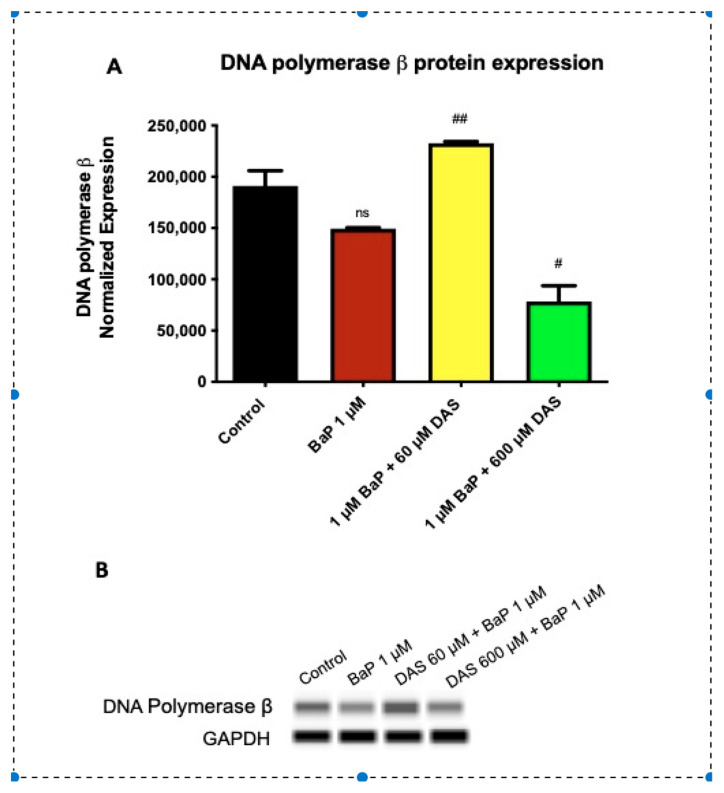
DAS and/or BaP effect on DNA Polymerase β (POLβ) expression in normal breast epithelial cells (MCF-10A) determined by Western analysis with automatic capillary electrophoresis. POLβ expression is an indicator of base excision repair. (**A**) A graph of normalized protein expression of POLβ measured by densitometry. (**B**) Electrophoretic bands represent the protein expression 24 hr-post treatment for POLβ expression. A statistically significant difference of DAS CoTx compared to BaP treatment alone was evaluated by one-way ANOVA followed by Dunnett’s multiple comparison tests. In triplicate, ±SEM. # indicates *p* < 0.05, ## indicates *p* < 0.01 compared to BaP, and ns indicates *p* > 0.05 when compared to control.

BaP produces oxidative DNA damage due to ROS generation. The DNA repair enzyme POLβ can be induced to repair the oxidative damage. POL β expression was evaluated for changes in protein expression due to treatment with BaP (1 μM) and/or DAS (60 or 600 μM). Following 24 h treatment, MCF-10A cells were harvested and lysed using a protease cocktail with lysis buffer. Protein expression was measured using Western analysis by automated capillary electrophoresis. The protein expression of all treatments was normalized using GAPDH loading control. All treatments were compared to the 1 μM BaP treatment (Figure 7A,B). Exposure to 1 μM BaP reduced POLβ expression when compared to the control. Co-treatment with 60 μM DAS and 1 μM BaP significantly (*p* < 0.001) increased the DNA polymerase expression when compared to 1 μM BaP alone, while CoTx with 600 μM DAS and BaP decreased expression (Figure 7A,B).

## 4. Discussion

Garlic has been evaluated for its chemopreventive properties in vitro, in vivo animal, and epidemiological research [20,21]. DAS is one of several garlic organosulfur compounds produced when the garlic clove’s integrity is altered by chopping, crushing, or mashing [22]. DAS has been shown to inhibit PhIP-induced carcinogenesis by inhibiting DNA strand breaks and lipid peroxidation in vitro [18,19] and inhibiting the formation of diethylstilbestrol (DES)-induced DNA adducts, oxidation, and lipid peroxidation in vivo [17,23,24]. DAS is one of several garlic organosulfur compounds with potential chemopreventive properties, though its mechanisms in inhibiting carcinogenesis have not been fully elucidated. In evaluating the role of pre-treatment versus co-treatment, DAS was shown to be more effective in inhibiting N-nitroso methyl benzylamine-induced esophageal tumors if administered during carcinogenesis initiation. Still, it was not as effective once the tumor had formed [25]. Studies conducted by Singh and Shukla [26] indicated that DAS was more effective in inhibiting BaP-induced skin carcinogenesis when applied 1 h after BaP exposure than a pre-treatment 1 h before. Additionally, the opposite was true for DMBA-induced skin carcinogenesis [27]. Therefore, DAS may be more effective in cancer prevention in different tissues and cell lines as pre-treatment, concurrent treatment, or post-induction therapy. DAS treatment must be evaluated through different treatment times relative to the carcinogenic initiator to fully understand DAS as a potential chemopreventive agent. 

The focus of this study was to evaluate how DAS inhibits early induced carcinogenesis activities such as alterations in cell viability and cell cycle, the formation of damaging ROS that can lead to DNA damage, and DNA strand breaks as a biomarker of DNA damage that can lead to mutations and carcinogenesis, through pre-and co-treatment of MCF-10A normal breast epithelial cells treated with BaP. At a concentration of one-micromole, BaP demonstrated potential carcinogenic activity in MCF-10A cells within 24 h of exposure as indicated by an increase in cell proliferation, transitioning cells into the G2/M phase of the cell cycle, and a significant increase in the formation of DNA-damaging ROS and induction of DNA strand breaks. 

DAS treatment alone has not been shown to alter cell viability in non-neoplastic cells. However, it has been effective in reducing carcinogen-induced cell toxicity [19,26,28]. DAS reduces cell viability in cancer cells through induction of apoptosis [10,29,30,31,32,33,34]. Only a few studies compare cell viability and apoptosis in normal and neoplastic cells treated with organosulfide compound (OSC). These studies have concluded that normal cells are more resistant to OSC-induced apoptosis than cancer cells [35,36,37] through an elusive selective mechanism [38]. Here we found that DAS co-treatment was effective in inhibiting BaP-induced cell proliferation at six hours, whereas the four-hour pre-treatment did not demonstrate the same effectiveness. These results may indicate findings in previous studies [38] that found the time of administration of the DAS concerning BaP exposure. Further research is needed to validate these findings. 

DAS is minimally active or not significant in altering cell cycle responses in cells. Compared to other garlic OSCs, DAS has not demonstrated effectiveness in inducing cell cycle arrest in prostate, colon, or liver cancer cells [39,40,41,42]. DAS, however, was shown to increase the accumulation of anaplastic thyroid carcinoma cells in the G2/M phase [33]. Our study presents novel research evaluating DAS’s role in inhibiting carcinogenic-induced G2/M phase increase in a normal breast cell line. Jeffy et al. [43] and Wang et al. [44] have demonstrated that BaP is more effective in inducing BaP-induced G2/M cell cycle accumulations after more than 24 h of prolonged exposure, indicating that DAS inhibits these changes. 

As a procarcinogen, BaP requires metabolic activation to produce its carcinogenic effects. Human mammary epithelial cells (HMEC) have been shown to metabolize BaP in vitro by preferentially biotransforming BaP to BPDE as the ultimate carcinogen [45]. During the metabolic process, reactive intermediates are produced that can cause DNA damage through ROS generation, resulting in oxidative damage to nucleic acids and proteins [46,47]. ROS-induced oxidative stress and an accumulation of DNA damage have been shown in breast malignancies [48,49,50,51,52,53]. Oxidative damage to DNA and the inactivation of DNA repair enzymes are likely involved in the etiology of oxidant-mediated cancer [54]. BaP-induced ROS has been shown to suppress the total antioxidant capacity of primary breast tissue and has demonstrated a significant correlation between clustered DNA damage and chromosomal aberrations, inducing the transformation of normal breast cells to a cancerous phenotype [25,54,55,56].

In our study, BaP significantly induced ROS and hydrogen peroxides (H_2_O_2_) in the extracellular medium, which are possible precursors for lipid peroxidation and oxidative damage. Most ROS have a short half-life and cause damage primarily locally. However, H_2_O_2_ is more persistent and can cause damage away from the source of origin [57,58]. In this study, all DAS pre- and concurrent treatments were effective in attenuating BaP-induced aqueous peroxides. The 6 μM DAS concurrent treatment with BaP-induced an increase in peroxide formation, which may be a precursor to garlic organosulfur compound-induced apoptosis [59]. However, we did not evaluate apoptosis in this study. Future research, including extended exposures (48, 72, 96 h), would be needed to elucidate this possibility. DAS has been shown to induce lipid peroxides in MCF-10A cells [18]. However, DAS has not significantly increased ROS production in cancer cells, leading to apoptosis of the neoplastic cells. It has been theorized that DAS may not be a primary inducer of ROS due to its carbon-sulfur-bound monosulfides, which are not cleaved by reducing agents. Thus, thiol formation does not occur, preventing significant ROS generation seen in other OSCs in cancer cells [60]. Through research presented in this study and conducted by our lab group, DAS has been shown to induce ROS in the form of both aqueous and lipid peroxides in normal cells and effectively reduce carcinogen-induced free radical induction [18,23].

Kryston et al. [58] suggest that persistent or chronic oxidative stress and damage significantly affect carcinogenesis. The use of oxidatively generated DNA damage is a likely biomarker for cancer. BaP has been previously shown to increase DNA strand breaks in human breast milk cells [61] and induce DNA single and double-strand breaks in primary breast epithelial cells [56]. Here, we have demonstrated that BaP significantly induces DNA strand breaks at a likely physiological concentration of 1 μM in a normal breast epithelial cell line. DAS at all concentrations through both pre-and concurrent treatment methods attenuated the BaP-induced DNA damage. The 6 μM DAS co-treatment was the least effective inhibitor of BaP-induced DNA damage than 60 and 600 μM DAS, and this is likely due to its initiation of ROS at the lower concentration. Previous studies have shown that increasing concentrations of DAS attenuate the mean Olive tail moment of MCF-10A cells, thus indicating DAS-induced DNA repair [18,19]. Additionally, Yu et al. [62] have determined that DAS does not form DNA adducts, leading to mutations. This data provides evidence that the garlic organosulfur compound DAS is an effective inhibitor of BaP-induced DNA strand breaks.

The literature has not shown a significant evaluation of garlic OSCs in the inhibition of DNA strand breaks through activation of DNA repair. There are five major DNA repair pathways, base excision repair (BER), nucleotide excision repair (NER), and mismatch repair (MMR), homologous recombination (HR), and non-homologous end-joining (NHEJ) [63]. DNA repair enzymes play an important role in maintaining genomic integrity [64]. In BER, proteins remove and replace damaged DNA bases, often formed during endogenous oxidative damage and hydrolytic decay of DNA, including single-strand breaks. The major oxidative base lesion formed due to the hydroxylation of the guanine C-8 residue is the saturated imidazole ring 7,8 dihydro-8-oxoguanine (8-oxo-G). This leads to the incorrect pairing of 8-oxo-guanine with adenine instead of cytosine, which may lead to the development of mutations and secondary DNA lesions [64]. In this study, exposure to 1 μM BaP in these MCF-10A cells caused a significant increase (*p* < 0.05) in 8-OHdG, an indicator of oxidative DNA damage and oxidative stress. Researchers have demonstrated that polycyclic aromatic hydrocarbons (PAHs) such as BaP damage DNA by developing DNA adducts and oxidative lesions following exposure [65]. Co-treatment with DAS attenuated BaP-induced increases in 8-OHdG levels in normal epithelial cells, indicating a decrease in oxidative DNA damage and oxidative stress. Research performed by Martinez-Gil et al. [66] showed that the antioxidants N-acetylcysteine and diallyl sulfide reduced ethanol-induced increases in CYP2E1 expression and superoxide anions, indicative of a decrease in oxidative stress. These results supported the DAS-induced reduction in oxidative stress in the data presented in this study and a previous study performed by Liu et al. [67] that found DAS attenuated ethanol-induced increases in malonaldehyde (a by-product of lipid peroxidation), lactate dehydrogenase (LDH), and aspartate aminotransferase (AST) in human hepatocytes. 

DNA polymerase X (POLX) enzymes are involved in the synthesis of short DNA segments rather than the replication of entire chromosomes [68]. DNA polymerases synthesize DNA with high accuracy and fidelity [69]. BER utilizes pOLβ to repair nuclear DNA damage caused by normal metabolism or environmental stressors, including ionizing radiation, oxidizing chemicals, or smoking [70]. POLβ performs small gap filling in BER to replace the previously removed damaged 8-oxo-G with the nucleoside base guanine [71]. 

The novel research conducted in this study demonstrated that BaP inhibited the protein expression of DNA polymerase β, which was attenuated by the garlic OSCs. In this study, DNA Pol β protein expression was reduced following 24-hour exposure to 1 μM BaP. CoTx of 1 μM BaP/ 60 μM DAS caused an increase in DNA Pol β in response at the same time point, while 24-h exposure to CoTx of BaP with 600 μM DAS caused a decrease in DNA Pol β. The increase in DNA Pol β in response to the 60 μM DAS CoTx are supported by the work of Yang et al. [72], showed that the SOD activity of wild type Polβ cells was significantly lower than Polβ null cells or Polβ overexpressing cells. Wu et al. [73] found that the DNA in Polβ null cells was more prone to damage and less likely to be repaired, while Polβ overexpressing cells had less DNA damage and were more likely to undergo DNA repair. The induction of POLβ with 60 μM DAS correlates well with the reduction of 8-OHdG levels at the same concentration of DAS. When examining the lipid peroxidation and oxidative DNA damage results in conjunction with the 600 μM DAS CoTx, there may be another mechanism involved in the repair of BaP-induced repair. These studies suggest that DNA Polβ may play a role in protection from the genotoxicity and genetic instability induced by BaP at certain concentrations. Furthermore, the normal expression level of DNA polβ may be indispensable to the maintenance of genome stability [64].

## 5. Conclusions

The results obtained indicate that DAS effectively inhibited BaP-induced cell proliferation, cell cycle transitions, ROS, and DNA damage in an MCF-10A cell line. Additionally, the obtained results provide more experimental evidence for garlic’s antitumor abilities and corroborate many epidemiological studies regarding the association between the increased intake of garlic and the reduced risk of several types of cancer. Future studies must be performed to gain further insight into the role of garlic in DNA damage and repair in breast cancer prevention.

## Data Availability

All data generated or analyzed during this study are included in this published article.

## References

[1] ACS (2021). Cancer Facts and Figures 2019–2020.

[2] Siegel R.L., Miller K.D., Jemal A. (2020). Cancer statistics 2020. CAN Cancer J. Clin..

[3] Anders C.K., Carey L.A. (2009). Biology, Metastatic Patterns, and Treatment of Patients with Triple-Negative Breast Cancer. Clin. Breast Cancer.

[4] Mendonca P., Alghamdi S., Messeha S., Soliman K.F.A. (2021). Pentagalloyl glucose inhibits TNF-α-activated CXCL1/GRO-α expression and induces apoptosis-related genes in triple-negative breast cancer cells. Sci. Rep..

[5] Cena H., Calder P.C. (2020). Defining a Healthy Diet: Evidence for The Role of Contemporary Dietary Patterns in Health and Disease. Nutrients.

[6] Trio P.Z., You S., He X., He J., Sakao K., Hou D.X. (2014). Chemopreventive functions and molecular mechanisms of garlic organosulfur compounds. Food Funct..

[7] Omar S.H., Al-Wabel N.A. (2010). Organosulfur compounds and possible mechanism of garlic in cancer. Saudi Pharm. J..

[8] Ip C., Lisk D.J., Stoewsand G.S. (1992). Mammary cancer prevention by regular garlic and selenium-enriched garlic. Nutr. Cancer.

[9] Schaffer E.M., Liu J.Z., Green J., Dangler C.A., Milner J.A. (1996). Garlic and associated allyl sulfur components inhibit N-methyl-N-nitrosourea induced rat mammary carcinogenesis. Cancer Lett..

[10] Rao P.S., Midde N.M., Miller D.D., Chauhan S., Kumar A., Kumar S. (2015). Diallyl Sulfide: Potential Use in Novel Therapeutic Interventions in Alcohol, Drugs, and Disease Mediated Cellular Toxicity by Targeting Cytochrome P450 2E1. Curr. Drug Metab..

[11] Yu T.-H., Wu C.-M., Liou Y.-C. (1989). Volatile Compounds from Garlic. J. Agric. Food Chem..

[12] Minami T., Boku T., Inada K., Morita M., Okazaki Y. (1989). Odor components of human breath after ingestion of grated raw garlic. J. Food Chem..

[13] Shukla Y., Arora A., Singh A. (2002). Antitumorigenic potential of diallyl sulfide in Ehrlich ascites tumor bearing mice. Biomed. Environ. Sci..

[14] Surh Y.J., Lee R.C., Park K.K., Mayne S.T., Liem A., Miller J.A. (1995). Chemoprotective effects of capsaicin and diallyl sulfide against mutagenesis or tumorigenesis by vinyl carbamate and N-nitrosodimethylamine. Carcinogenesis.

[15] Tadi P.P., Teel R.W., Lau B.H. (1991). Organosulfur compounds of garlic modulate mutagenesis, metabolism, and DNA binding of aflatoxin B1. Nutr. Cancer.

[16] Hageman G.J., van Herwijnen M.H., Schilderman P.A., Rhijnsburger E.H., Moonen E.J., Kleinjans J.C. (1997). Reducing effects of garlic constituents on DNA adduct formation in human lymphocytes in vitro. Nutr. Cancer.

[17] Green M., Wilson C., Newell O., Sadrud-Din S., Thomas R. (2005). Diallyl sulfide inhibits diethylstilbesterol-induced DNA adducts in the breast of female ACI rats. Food Chem. Toxicol..

[18] Wilson C., Aboyade-Cole A., Newell O., Darling-Reed S., Oriaku E., Thomas R. (2007). Diallyl sulfide inhibits PhIP-induced DNA strand breaks in normal human breast epithelial cells. Oncol. Rep..

[19] Aboyade-Cole A., Darling-Reed S., Oriaku E., McCaskill M., Thomas R. (2008). Diallyl sulfide inhibits PhIP-induced cell death via the inhibition of DNA strand breaks in normal human breast epithelial cells. Oncol. Rep..

[20] Nkrumah-Elie Y.M., Reuben J.S., Hudson A.M., Taka E., Badisa R., Ardley T., Israel B., Sadrud-Din S.Y., Oriaku E.T., Darling-Reed S.F. (2012). The attenuation of early benzo(a)pyrene-induced carcinogenic insults by diallyl disulfide (DADS) in MCF-10A cells. Nutr. Cancer.

[21] Nkrumah-Elie Y.M., Reuben J.S., Hudson A., Taka E., Badisa R., Ardley T., Israel B., Sadrud-Din S.Y., Oriaku E., Darling-Reed S.F. (2012). Diallyl trisulfide as an inhibitor of benzo(a)pyrene-induced precancerous carcinogenesis in MCF-10A cells. Food Chem. Toxicol..

[22] Block E. (1985). The chemistry of garlic and onions. Sci. Am..

[23] Gued L.R., Thomas R.D., Green M. (2003). Diallyl sulfide inhibits diethylstilbestrol-induced lipid peroxidation in breast tissue of female ACI rats: Implications in breast cancer prevention. Oncol. Rep..

[24] Thomas R.D., Green M.R., Wilson C., Sadrud-Din S. (2004). Diallyl sulfide inhibits the oxidation and reduction reactions of stilbene estrogens catalyzed by microsomes, mitochondria and nuclei isolated from breast tissue of female ACI rats. Carcinogenesis.

[25] Wargovich M.J., Woods C., Eng V.W., Stephens L.C., Gray K. (1988). Chemoprevention of N-nitrosomethylbenzylamine-induced esophageal cancer in rats by the naturally occurring thioether, diallyl sulfide. Cancer Res..

[26] Siriwardhana N., Wang H.C. (2008). Precancerous carcinogenesis of human breast epithelial cells by chronic exposure to benzo[a]pyrene. Mol. Carcinog..

[27] Singh A., Shukla Y. (1998). Antitumour activity of diallyl sulfide on polycyclic aromatic hydrocarbon-induced mouse skin carcinogenesis. Cancer Lett..

[28] Singh A., Shukla Y. (1998). Antitumour activity of diallyl sulfide in two-stage mouse skin model of carcinogenesis. Biomed. Environ. Sci..

[29] Sheen L.Y., Wu C.C., Lii C.K., Tsai S.J. (2001). Effect of diallyl sulfide and diallyl disulfide, the active principles of garlic, on the aflatoxin B(1)-induced DNA damage in primary rat hepatocytes. Toxicol. Lett..

[30] Wu P.P., Chung H.W., Liu K.C., Wu R.S., Yang J.S., Tang N.Y., Lo C., Hsia T.C., Yu C.C., Chueh F.S. (2011). Diallyl sulfide induces cell cycle arrest and apoptosis in HeLa human cervical cancer cells through the p53, caspase- and mitochondria-dependent pathways. Int. J. Oncol..

[31] Shin H.A., Cha Y.Y., Park M.S., Kim J.M., Lim Y.C. (2010). Diallyl sulfide induces growth inhibition and apoptosis of anaplastic thyroid cancer cells by mitochondrial signaling pathway. Oral Oncol..

[32] Wang H.C., Yang J.H., Hsieh S.C., Sheen L.Y. (2010). Allyl sulfides inhibit cell growth of skincancer cells through induction of DNA damage mediated G2/M arrest and apoptosis. J. Agric. Food Chem..

[33] Truong D., Hindmarsh W., O’Brien P.J. (2009). The molecular mechanisms of diallyl disulfide and diallyl sulfide induced hepatocyte cytotoxicity. Chem. Biol. Interact..

[34] Sriram N., Kalayarasan S., Ashokkumar P., Sureshkumar A., Sudhandiran G. (2008). Diallyl sulfide induces apoptosis in Colo 320 DM human colon cancer cells: Involvement of caspase-3, NF-kappaB, and ERK-2. Mol. Cell Biochem..

[35] Hong Y.S., Ham Y.A., Choi J.H., Kim J. (2000). Effects of allyl sulfur compounds and garlic extract on the expression of Bcl-2, Bax, and p53 in non small cell lung cancer cell lines. Exp. Mol. Med..

[36] Karmakar S., Banik N.L., Patel S.J., Ray S.K. (2007). Garlic compounds induced calpain and intrinsic caspase cascade for apoptosis in human malignant neuroblastoma SH-Sy5Y cells. Apoptosis.

[37] Kim Y.A., Xiao D., Xiao H., Powolny A.A., Lew K.L., Reilly M.L., Zeng Y., Wang Z., Singh S.V. (2007). Mitochondria-mediated apoptosis by diallyl trisulfide in human prostate cancer cells is associated with generation of reactive oxygen species and regulated by Bax/Bak. Mol. Cancer Ther..

[38] Powolny A.A., Singh S.V. (2008). Multitargeted prevention and therapy of cancer by diallyl trisulfide and related Allium vegetable-derived organosulfur compounds. Cancer Lett..

[39] Ansar S., Iqbal M., AlJameil N. (2014). Diallyl sulphide, a component of garlic, abrogates ferric nitrilotriacetate-induced oxidative stress and renal damage in rats. Hum. Exp. Toxicol..

[40] Xiao D., Srivastava S.K., Lew K.L., Zeng Y., Hershberger P., Johnson C.S., Trump D.L., Singh S.V. (2003). Allyl isothiocyanate, a constituent of cruciferous vegetables, inhibits proliferation of human prostate cancer cells by causing G2/M arrest and inducing apoptosis. Carcinogenesis.

[41] Xiao D., Herman-Antosiewicz A., Antosiewicz J., Xiao H., Brisson M., Lazo J.S., Singh S.V. (2005). Diallyl trisulfide-induced G(2)-M phase cell cycle arrest in human prostate cancer cells is caused by reactive oxygen species-dependent destruction and hyperphosphorylation of Cdc 25C. Oncogene.

[42] Xiao D., Pinto J.T., Gundersen G.G., Weinstein I.B. (2005). Effects of a series of organosulfur compounds on mitotic arrest and induction of apoptosis in colon cancer cells. Mol. Cancer Ther..

[43] Wu C.C., Chung J.G., Tsai S.J., Yang J.H., Sheen L.Y. (2004). Differential effects of allyl sulfides from garlic essential oil on cell cycle regulation in human liver tumor cells. Food Chem. Toxicol..

[44] Jeffy B.D., Chen E.J., Gudas J.M., Romagnolo D.F. (2000). Disruption of cell cycle kinetics by benzo[a]pyrene: Inverse expression patterns of BRCA-1 and p53 in MCF-7 cells arrested in S and G2. Neoplasia.

[45] Wang Z., Qi Y., Chen Q., Yang D., Tang S., Jin X., Gao J., Fu J., Zhou Z., Wang J. (2009). Cyclin A is essential for the p53-modulated inhibition from benzo(a)pyrene toxicity in A549 cells. Toxicology.

[46] Leadon S.A., Stampfer M.R., Bartley J. (1988). Production of oxidative DNA damage during the metabolic activation of Benzo [a]pyrene in human mammary epithelial cells correlates with cell killing. Proc. Natl. Acad. Sci. USA.

[47] Jung D., Cho Y., Collins L.B., Swenberg J.A., Di Giulio R.T. (2009). Effects of benzo[a]pyrene on mitochondrial and nuclear DNA damage in Atlantic killifish (Fundulus heteroclitus) from a creosote-contaminated and reference site. Aquat. Toxicol..

[48] Rekhadevi P.V., Diggs D.L., Huderson A.C., Harris K.L., Archibong A.E., Ramesh A. (2014). Metabolism of the environmental toxicant benzo(a)pyrene by subcellular fractions of human ovary. Hum. Exp. Toxicol..

[49] Gollapalle E., Wang R., Adetolu R., Tsao D., Francisco D., Sigounas G., Georgakilas A.G. (2007). Detection of oxidative clustered DNA lesions in X-irradiated mouse skin tissues and human MCF-7 breast cancer cells. Radiat. Res..

[50] Curtis C.D., DThorngren L., Nardulli A.M. (2010). Immunohistochemical analysis of oxidative stress and DNA repair proteins in normal mammary and breast cancer tissues. BMC Cancer.

[51] Matsui A., Ikeda T., Enomoto K., Hosoda K., Nakashima H., Omae K., Watanabe M., Hibi T., Kitajima M. (2000). Increased formation of oxidative DNA damage, 8-hydroxy-2′-deoxyguanosine, in human breast cancer tissue and its relationship to GSTP1 and COMT genotypes. Cancer Lett..

[52] Musarrat J., Arezina-Wilson J., Wani A.A. (1996). Prognostic and aetiological relevance of 8-hydroxyguanosine in human breast carcinogenesis. Eur. J. Cancer.

[53] Nyaga S.G., Jaruga P., Lohani A., Dizdaroglu M., Evans M.K. (2007). Accumulation of oxidatively induced DNA damage in human breast cancer cell lines following treatment with hydrogen peroxide. Cell Cycle.

[54] Atukeren P., Yavuz B., Soydinc H.O., Purisa S., Camlica H., Gumustas M.K., Balcioglu I. (2010). Variations in systemic biomarkers of oxidative/nitrosative stress and DNA damage before and during the consequent two cycles of chemotherapy in breast cancer patients. Clin. Chem. Lab. Med..

[55] Tapiero H. (2004). Influence of alcohol consumption and smoking habits on human health. Biomed. Pharmacother..

[56] Caruso J.A., Reiners J.J., Emond J., Shultz T., Tainsky M.A., Alaoui-Jamali M., Batist G. (2001). Genetic alteration of chromosome 8 is a common feature of human mammary epithelial cell lines transformed in vitro with benzo[a]pyrene. Mutat. Res..

[57] Sigounas G., Hairr J.W., Cooke C.D., Owen J.R., Asch A.S., Weidner D.A., Wiley J.E. (2010). Role of benzo[alpha]pyrene in generation of clustered DNA damage in human breast tissue. Free Radic. Biol. Med..

[58] Sokolov M.V., Dickey J.S., Bonner W.M., Sedelnikova O.A. (2007). gamma-H2AX in bystander cells: Not just a radiation-triggered event, a cellular response to stress mediated by intercellular communication. Cell Cycle.

[59] Kryston T.B., Georgiev A.B., Pissis P., Georgakilas A.G. (2011). Role of oxidative stress and DNA damage in human carcinogenesis. Mutat. Res..

[60] Wu X., Kassie F., Mersch-Sundermann V. (2005). Induction of apoptosis in tumor cells by naturally occurring sulfur-containing compounds. Mutat. Res..

[61] Iciek M., Kwiecien I., Wlodek L. (2009). Biological properties of garlic and garlic-derived organosulfur compounds. Environ. Mol. Mutagen..

[62] Martin F.L., Cole K.J., Williams J.A., Millar B.C., Harvey D., Weaver G., Grover P.L., Phillips D.H. (2000). Activation of genotoxins to DNA-damaging species in exfoliated breast milk cells. Mutat. Res..

[63] Yu F.L., Bender W., Fang Q., Ludeke A., Welch B. (2003). Prevention of chemical carcinogen DNA binding and inhibition of nuclear RNA polymerase activity by organosulfur compounds as the possible mechanisms for their anticancer initiation and proliferation effects. Cancer Detect. Prev..

[64] Chattergee N., Walker G.C. (2017). Mechanisms of DNA Damage, Repair, and Mutagenesis. Environ. Mol. Mutagen..

[65] Wang R., Wenjing H., Pan L., Boldogh I., Ba X. (2018). The role of base excision repair enzyme OGG1 in gene expression. Cell. Mol. Life Sci..

[66] Mordukhovich I., Beyea J., Herring A.H., Hatch M., Stellman S.D., Teltelbaum S.L., Richardson D.B., Millikan R.C., Engel L.S., Shantakumar S. (2016). Polymorphisms in DNA repair genes, traffic-related polycyclic aromatic hydrocarbon exposure and breast cancer incidence. Int. J. Cancer.

[67] Martinez-Gil N., Vidal-Gil L., Flores-Bellvor M., Maista R., Sancho-Pelluz J., Diaz-Llopis M., Barcia J.M., Romero F.J. (2020). Ethanol-Induced Oxidative Stress modifies Inflammation and Angiogenesis Biomarkers in Retinol Pigment Epithelial Cells (ARPE-19): Role of CYP2E1 and its inhibition by Antioxidants. Antioxidants.

[68] Liu L.-G., Yan H., Yao P., Zheng W., Zou L.-J., Sang F.-F., Li K., Sin X.-F. (2005). CYP2E1-dependent hepatotoxicity and oxidative damage after ethanol administration in human primary hepatocytes. World J. Gastroenterol..

[69] Uchiyama Y., Takeuchi P., Kodera H., Sakaguchi K. (2009). Distribution and roles of X-family DNA polymerases in eukaryotes. Biochimie.

[70] Beard W.A., Wilson S.H. (2014). Structure and mechanism of DNA polymerase beta. Biochemistry.

[71] Souliotis V.L., Vlachogiannis N.I., Pappa M., Argyriou A., Ntouros P.A., Sfikakis P.P. (2020). DNA damage response and oxidative stress in systemic autoimmunity. Int. J. Mol. Sci..

[72] Yang M., Wu M., Cui J., Chen C., Zhang Z.-Z. (2011). Effects of DNA polymerase beta on the genotoxicity and genetic instability induced benzo(a)pyrene. Zhonghue Lao Dong Wei Sheng Zhi Ye Bing Za Zhi.

[73] Wu M., Lai Y., Zhang Z. (2010). [Effect of DNA polymerase beta on repair of DNA damage induced by benzo(a)pyrene]. Wei Sheng Yan Jiu.

